# Pituitary Involvement in Granulomatosis With Polyangiitis: Report of 9 Patients and Review of the Literature

**DOI:** 10.1097/MD.0000000000000748

**Published:** 2015-04-24

**Authors:** Audrey De Parisot, Xavier Puéchal, Corinne Langrand, Gerald Raverot, Helder Gil, Laurent Perard, Guillaume Le Guenno, Sabine Berthier, Olivier Tschirret, Jean Paul Eschard, Stephane Vinzio, Loïc Guillevin, Pascal Sève, and the French Vasculitis Study Group

**Affiliations:** From the Department of Internal Medicine, Hôpital de La Croix-Rousse and University Claude Bernard Lyon 1, 103 Grande rue de la Croix-Rousse 69317 Lyon Cedex 04 (ADP, PS); Department of Internal Medicine, Centre de Référence des maladies systémiques autoimmunes rares, Hôpital Cochin, University Paris-Descartes, Assistance Publique – Hôpitaux de Paris, Paris (XP, LG); Department of Endocrinology, Groupement Hospitalier Est and University Claude Bernard Lyon 1, 59 Boulevard Pinel 69677 Bron Cedex, Lyon (CL, GR); Department of Internal Medicine, Centre Hospitalier Régional Universitaire de Besançon, 2 Place Saint-Jacques, Besançon (HG); Department of Internal Medicine, Hôpital Edouard Herriot and University Claude Bernard Lyon 1, Lyon (LP); Department of Internal Medicine, Hôpital de Hautepierre, 1 Avenue Molière, Strasbourg (LG); Department of Internal Medicine, Centre Hospitalier Universitaire de Dijon, 14 rue Paul Gaffarel, Dijon (SB); Department of Neuroradiology, Groupement Hospitalier Est and University Claude Bernard Lyon 1, 59 Boulevard Pinel 69677 Bron Cedex, Lyon (OT); Department of Rheumatology, Hôpital de la Maison Blanche, 45 rue Cognacq-Jay 51092 Reims Cedex (JPE); and Department of Internal Medicine, Groupe Hospitalier Mutualiste de Grenoble, 8 Rue Docteur Calmette 38000 Grenoble, France (SV).

## Abstract

Pituitary dysfunction is a rare manifestation of granulomatosis with polyangiitis (GPA) (Wegener). The main aim of this multicenter retrospective study was to describe the characteristics and outcomes of pituitary manifestations in patients with GPA included in the French Vasculitis Study Group database.

Among the 819 GPA patients included in the database, 9 (1.1%) had pituitary involvement. The median age at diagnosis of GPA and pituitary involvement was 46 and 50.8 years, respectively. Pituitary involvement was present at onset of GPA in 1 case and occurred later in 8 patients after a median follow up of 58.5 months. When pituitary dysfunction occurred, 8 patients had active disease at other sites including ENT (n = 6), eye (n = 4), or central nervous system (n = 3) involvement. The most common hormonal dysfunctions were diabetes insipidus (n = 7) and hypogonadism (n = 7). Magnetic resonance imaging was abnormal in 7 patients. The most common lesions were an enlargement of the pituitary gland, thickening of the pituitary stalk, and loss of posterior hypersignal on T1-weighed images. All patients were treated with corticosteroid therapy and 8 patients received immunosuppressive agents for the pituitary involvement, including cyclophosphamide (n = 3), rituximab (n = 2), and methotrexate (n = 3). After a median follow-up of 9.2 years, GPA was in complete remission in 7 patients, but 8 patients were still under hormone replacement therapy. Among the 5 patients who had a subsequent MRI, 2 had complete resolution of pituitary lesions.By combining our study and the literature review, the frequency of hypogonadism and diabetes insipidus, among the patients with pituitary dysfunction, can be estimated at 78% and 71% respectively. Despite a high rate of systemic disease remission on maintenance therapy, 86% of the patients had persistent pituitary dysfunction. The patients who recovered from pituitary dysfunction had all been treated by cyclophosphamide.

Pituitary disease in GPA occurs mostly several months or years after diagnosis. There is no correlation between hormonal, radiologic, and systemic outcome. Although immunosuppressive drugs improve the systemic disease, hormonal deficiencies usually persist. It is therefore important to shorten diagnostic delays and treat these patients early in the course of disease before irreversible damage occur.

## INTRODUCTION

Granulomatosis with polyangiitis (GPA) is a systemic disease characterized by necrotizing small-vessel vasculitis of unknown etiology. It is often associated with anti-neutrophil cytoplasmic antibodies (ANCAs). It generally involves the upper and lower respiratory tracts, the kidneys, and the ear, nose, and throat (ENT). However, any organ or tissue can potentially be affected. The nervous system is involved in 22% to 54% of cases.^1^ Peripheral neuropathies and cranial nerve palsies are the most common forms. Central nervous system (CNS) involvement is much less common and is estimated to occur in approximately 10% of patients.^2^

Pituitary dysfunction (PD) is a rare manifestation of GPA. The only published series about PD involvement in GPA included 8 patients and suggested that gonadotropin deficiency and diabetes insipidus were the most frequent manifestation of the disease.^3^ Furthermore, this study showed that pituitary hormone deficiency may persist despite adequate response of systemic disease and resolution of head imaging findings.

We present here a retrospective series of 9 patients with pituitary involvement included in the French Vasculitis Study Group (FVSG) database and a literature review. The aims of this study were: to describe the clinical and biological characteristics of patients with pituitary manifestations of GPA, and to assess the response to treatment and patient outcome.

## MATERIALS AND METHODS

### Patients

The FVSG computerized database contains data about patients diagnosed with polyarteritis nodosa, GPA, eosinophilic granulomatosis with polyangiitis, or microscopic polyangiitis; who satisfied the 1990 classification criteria by the American College of Rheumatology for GPA^4^ and/or the 2012 revised International Chapel Hill Consensus Conference Nomenclature of Vasculitides^5^; and who had been enrolled in one of the FVSG trials^6^ and/or referred to the Department of Internal Medicine at Avicenne Hospital (Bobigny, France) up to September 2003 or to Cochin Hospital (Paris, France) thereafter up to September 2007; data are updated regularly, at least every alternative year. Vasculitis diagnoses made before 1990 were reassessed by the investigators, who considered the entire follow-up of all patients. Patients who participated in prospective therapeutic trials gave their written informed consent for the collection and analysis of data for future ancillary studies. Other patients received oral and written information informing them of their unrestricted rights to ask for the deletion of their data. The FVSG database was reported to the Commission Nationale Informatique et Libertés at its inception in 1980. The patients were eligible for our study if they met the following criteria: patient older than 18 years at the time of the study; pituitary involvement; exclusion of other possible causes, including other granulomatous disorders; exclusion of any other cause of pituitary dysfunction.

### Data Collection

Clinical and laboratory data were collected by the same investigator (AP) using a standardized form. Data about medical history, onset of the disease, clinical symptoms, organ involvement, tissue biopsies, MRI imaging, and laboratory data including C-reactive protein (CRP), ANCAs, and response to therapy were recorded at diagnosis and at end of follow-up. There was no systematic screening for pituitary dysfunction. Patients were diagnosed with PD based on symptoms, laboratory data, and MRI imaging.

### Hormonal Assessment

Pituitary dysfunction was demonstrated by basal hormonal modifications (involving anterior and/or posterior pituitary), with or without specific clinical signs. Gonadotrophin axis was evaluated by serum measurements: follicule-stimulating hormone (FSH), luteinizing hormone (LH), estradiol, and testosterone. Gonadotrophin deficiency was defined as a low basal gonadal steroid concentration with concomitant low or normal gonadotropin levels. Thyrotropin axis was evaluated by serum measurements: thyroid-stimulating hormone (TSH), free thyroxine (FT4), and free triiodothyronine (FT3). Thyrotropin deficiency was defined as low FT4 (with low or normal FT3) without TSH elevation. Growth hormone axis (GH) was evaluated by a serum measurement of growth hormone and insulin-like growth factor-1 (IGF1). GH deficiency was suspected when IGF1 was below normal value. Corticotropin axis was evaluated by serum measurement: serum cortisol and adrenocorticotropic hormone (ACTH) at 8:00 am. ACTH deficiency was defined as low basal cortisol concentration at 8:00 am with low or inappropriately normal ACTH or as impaired cortisol stimulation after corticotropin test when available. If the patient was already under corticosteroid treatment for the GPA, the corticotropin axis was not evaluated. Prolactin (PRL) was measured and hyperprolactinemia was considered when prolactin blood level was higher than the upper value of the laboratory. Diabetes insipidus (DI) was defined as a polyuropolydipsic syndrome (urinary volume >3 L/day) improved by arginine vasopressin and/or a low urinary osmolarity persistent after a water deprivation test. Panhypopituitarism was defined as hormonal deficiency including FSH, LH, ACTH, TSH, GH, and DI.

### Radiologic Assessment

The evaluation included MRI examinations of the brain and sella turcica in all patients. The area of the hypothalamo-pituitary axis was investigated in both sagittal and coronal planes using a T1-weighted spoiled gradient-echo sequence before and after gadolinium administration and a T2 sequence. MRI was performed if clinical or biological signs were present at baseline and under treatment. Pituitary lesions were classified according to their localization, size, gadolinium enhancement, and extension.

### Assessment of Treatment Effectiveness

For each patient, the number of deficient axes was assessed at diagnosis of pituitary dysfunction and at the end of follow-up. The involvement of pituitary function was classified by axis: 5 axes for the anterior gland (gonadotropin, TSH, GH, ACTH, and PRL) and 1 for the posterior gland (diagnosis of DI). MRI findings were classified according to the sites of the lesions as described above.

### Literature Review

We performed a Medline search using the term “Wegener” or “Granulomatosis with polyangiitis” and “pituitary” or “hypothalamo pituitary” to identify all articles published online. Our systematic literature search was limited to the English and French language. The reference lists of all the articles were scanned for references not identified in the initial research. Only cases with well-documented clinical summaries and relevant information were included.

## RESULTS

### Patient Characteristics

Among the 819 patients with a diagnosis of GPA between 1963 and 2014 included in the FVSG database, 9 (1.1%) patients from 7 different French Centers (Croix Rousse Hospital, Lyon; Edouard Herriot Hospital, Lyon; Besançon Hospital, Besançon; Estaing Hospital, Clermond-Ferrand; Hautepierre Hospital, Strasbourg; Reims Hospital, Reims; Cochin Hospital, Paris) were identified as having pituitary dysfunction related to GPA. The clinical characteristics and results of the tests of all the 9 patients are summarized in Table 1. Due to the multicentric and retrospective nature of the study, patients were evaluated in different laboratories. Thus, the hormonal values were expressed in different units, with different normal ranges, which is why there is no table with the actual hormone values.

**TABLE 1:**
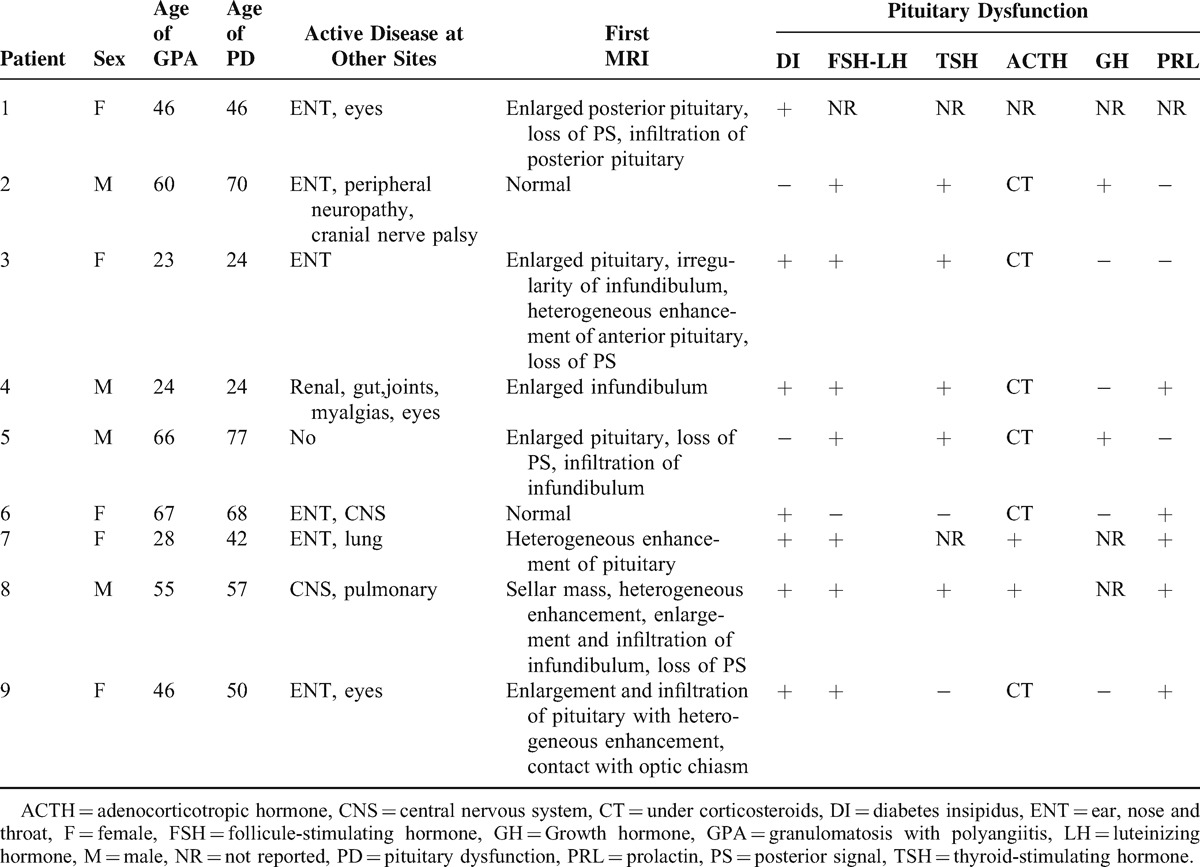
Clinical, Hormonal, Radiologic Features in Patients With HP Involvement in GPA

Five patients were female and 4 were male. The median age at diagnosis of GPA was 46 years (range: 23–67 years) and at diagnosis of PD 50.8 years (range: 24–77 years). PD was diagnosed after the diagnosis of GPA in 8 patients with a median of 58.5 months (range: 6–165 months) and the diagnosis was concomitant in 1 case. Median time to diagnosis of PD was 10.4 months (range: 1–36 months). All patients had a biopsy consistent with GPA and 7 were ANCA-positive, of whom 6 were PR3-ANCA-positive. One patient had a pituitary biopsy that showed a nonspecific hypophysitis without any granuloma. This patient was not tested for serum pituitary antibodies. During the course of GPA, upper respiratory tract manifestations were present in all patients but 1 (89%), pulmonary opacities in 7 (78%), glomerulonephritis in 3 (33.3%). Four patients had CNS involvement (3 meningitis, 1 cranial nerve palsy) (44.4%), and 4 patients had mono- or polyneuropathy.

At the time PD was diagnosed, all patients but 1 had active disease at other sites including: ENT (n = 6), eye (n = 4), CNS (n = 3), lung (n = 2), or kidney involvement (n = 1).

### Clinical Endocrine Features

All patients reported clinical symptoms of PD. Clinical manifestations were headaches (n = 6), vomiting (n = 2), polyuropolydipsia (n = 7), asthenia (n = 4), amenorrhea (n = 3), galactorrhea (n = 1), decreased libido (n = 1), muscular atrophy (n = 2), and decreased pilosity (n = 1).

### Hormonal Evaluation

An anterior pituitary dysfunction was reported in 8 patients and a posterior pituitary dysfunction in 7 patients. No patient had panhypopituitarism. Diabetes insipidus was the most common deficiency and was reported in 7 patients. It was associated with an anterior pituitary deficiency in all cases but 1. In this patient, it presented as the initial manifestation of GPA, but it was associated with ENT and eye involvement. Six patients underwent water deprivation tests and the remaining patient was diagnosed on the basis of polyuropolydipsia. Seven patients had hypogonadism, including 2 premenopausal patients who had previously been treated with cyclophosphamide. Five patients had TSH deficiency. Four patients had hyperprolactinemia (with lesions of the stalk on MRI for 2 of them). GH deficiency was diagnosed on the basis of low IGF1 in 2 patients. One patient had an ACTH deficiency based on a low 8 am cortisol. Corticotropin axis was not evaluated in 7 patients who were already treated by corticosteroids and was not reported in 1 case.

### Radiologic Features

**FIGURE 1:**
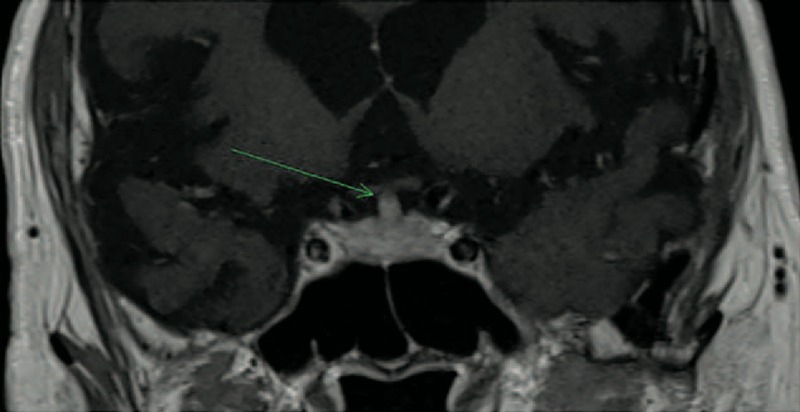
Patient 5 in 2014: coronal brain T1-weighted MR image after intravenous administration of gadolinium showing an enlargement of pituitary stalk (4 mm).

**FIGURE 2:**
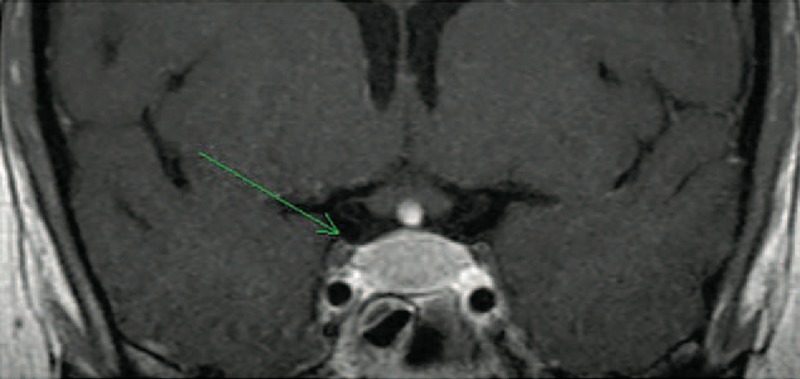
Patient 3 in 2007: coronal brain T1-weighted MR image after intravenous administration of gadolinium showing an enlargement and intense enhancement of pituitary.

**FIGURE 3:**
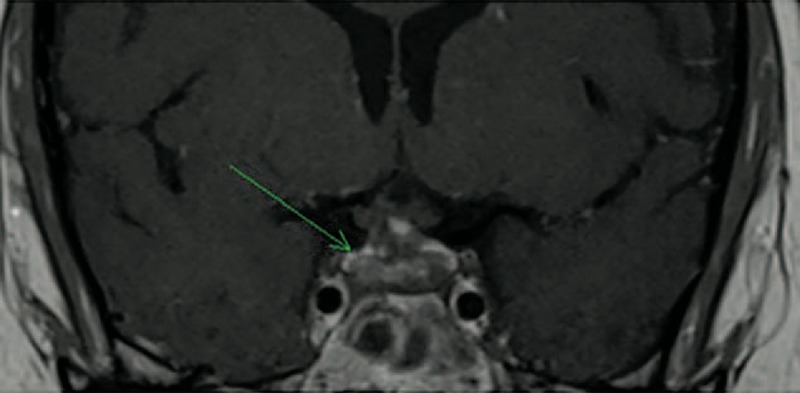
Patient 3 in 2011: coronal brain T1-weighted MR image after intravenous administration of gadolinium showing reduction in size of pituitary with heterogeneous enhancement.

All but two patients presented MRI abnormalities of the pituitary area (Figs. 1–3). The most common lesions were: enlargement of the pituitary gland or pseudo adenoma (n = 5), loss of posterior hypersignal on T1-weighed images (n = 4), thickening or infiltrative lesion of the pituitary stalk (n = 4) and infiltrative lesions or enhanced lesions by gadolinium of the pituitary gland (n = 4). Other lesions of the central nervous system included a thickening of the dura matter in one patient.

### Follow-Up and Outcome Under Treatment

Treatment of patients is summarized in Table 2. All patients were treated with corticosteroid therapy for the pituitary involvement and 3 of them received high doses of methylprednisolone intravenously (500 mg/day). All patients but 1 received immunosuppressive agents for the pituitary involvement including cyclophosphamide (n = 3), rituximab (n = 2), methotrexate (n = 3), infliximab (n = 3), intravenous immunoglobulin (IVIG) (n = 2), azathioprine (n = 2), mycophenolate mofetil (n = 1), and chlorambucil (n = 1). No patient underwent surgery.

**TABLE 2:**
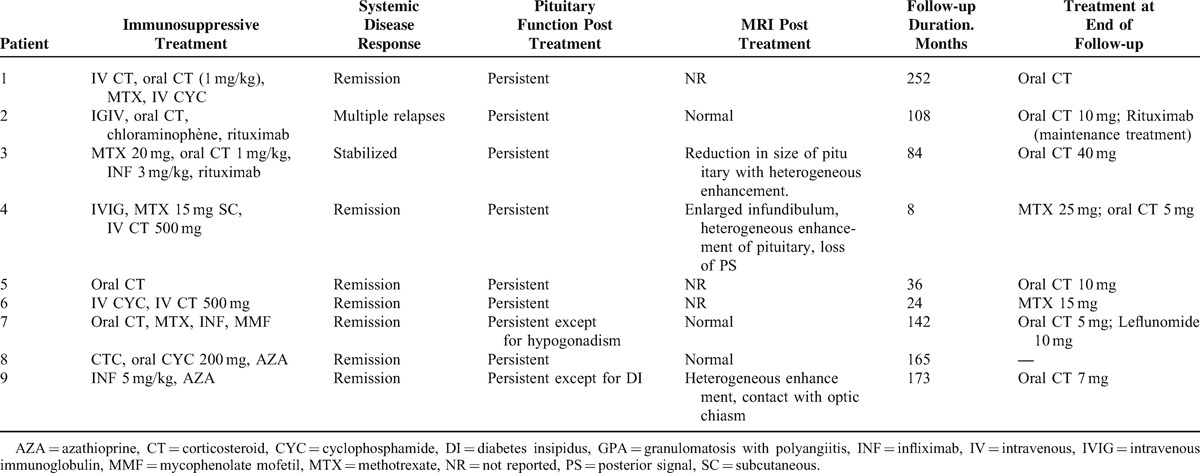
Treatment and Outcome of HP involvement in patients with GPA

The mean follow-up duration starting from the diagnosis of pituitary involvement was 9.2 years (range: 8 months–21 years). The systemic disease was considered in complete remission for 7 patients. One patient had disease activity controlled with methotrexate, IVIG, and corticosteroid therapy. The last patient presented multiple relapses due to ENT involvement. All patients but 2 (who were PR3-ANCA-positive) were ANCA-negative at the end of follow-up.

Hormone replacement therapy was initiated for 8 patients including desmopressin (n = 6), levothyroxine (n = 5), testosterone (n = 3), and oestroprogestative treatment (n = 1). At the end of follow-up, all these patients were still under hormone replacement therapy and additional deficiencies (thyreotropin and gonadotropin) were revealed for patient 3. Actually, 1 patient recovered from her desmopressin deficiency, but it was not reported whether it had initially been confirmed by a water deprivation test or not (Patient 7).

Of the 7 patients who had pituitary lesions before treatment, 5 had MRI evaluations of the Hypothalamo-Pituitary (HP) lesions during follow-up: 2 had complete regression, 1 had partial improvement, and 2 had no modification.

The outcome of pituitary lesions was not correlated with the course of hormonal status. At the end of follow-up, 7 patients remained glucocorticoid-dependent and 4 patients had maintenance treatment for the vasculitis including intermittent rituximab infusions (n = 1), methotrexate (n = 2), and leflunomide (n = 1).

### Literature Review

To date, only 1 case series^3^ and 34 case reports^7–35^ have been published on this rare involvement in GPA (Tables 3  Tables 4 ).^2,3,7–25,27–35^ The median age at diagnosis of PD was 38.6 years old (range: 13–71 years) and there was a female predominance (71%). The diagnosis of PD preceded the development of vasculitis in 3 patients (7.6%), was concomitant in 18 (46%), and occurred subsequently in 18 (46%). The most frequent organs involved during the course of GPA were ENT (90%), followed by lung in 43.5 %, CNS in 42.5%, and eye in 32.5%. ANCA were positive in 91.9% of patients with a perinuclear specificity in 58.8% of patients. The perinuclear or myeloperoxidase specificity was unknown in 41.2% of cases. When PD occurred, 25 of 34 patients had active disease at other sites including ENT (n = 14), lung (n = 9), eye (n = 7), CNS (n = 4), and kidney (n = 3).

**TABLE 3:**
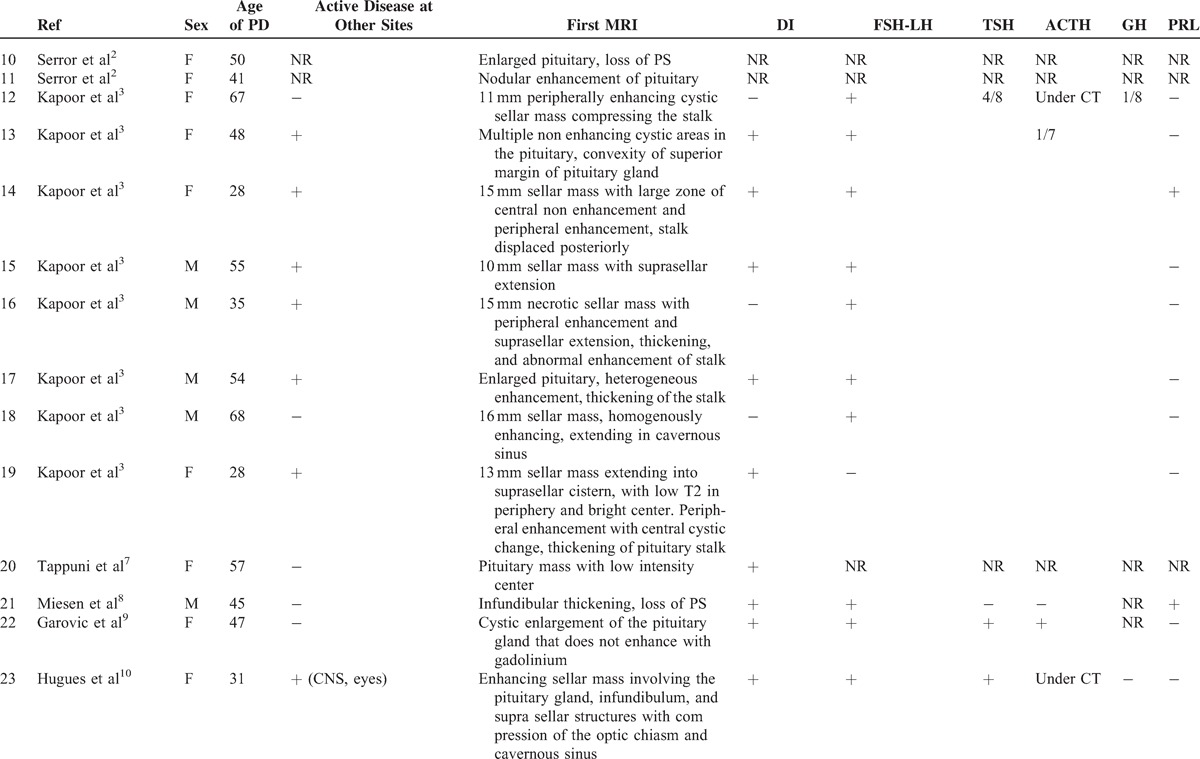
Clinical, Hormonal, Radiologic Features in patients With HP Involvement in GPA (Literature Review)

**TABLE 3 (Continued):**
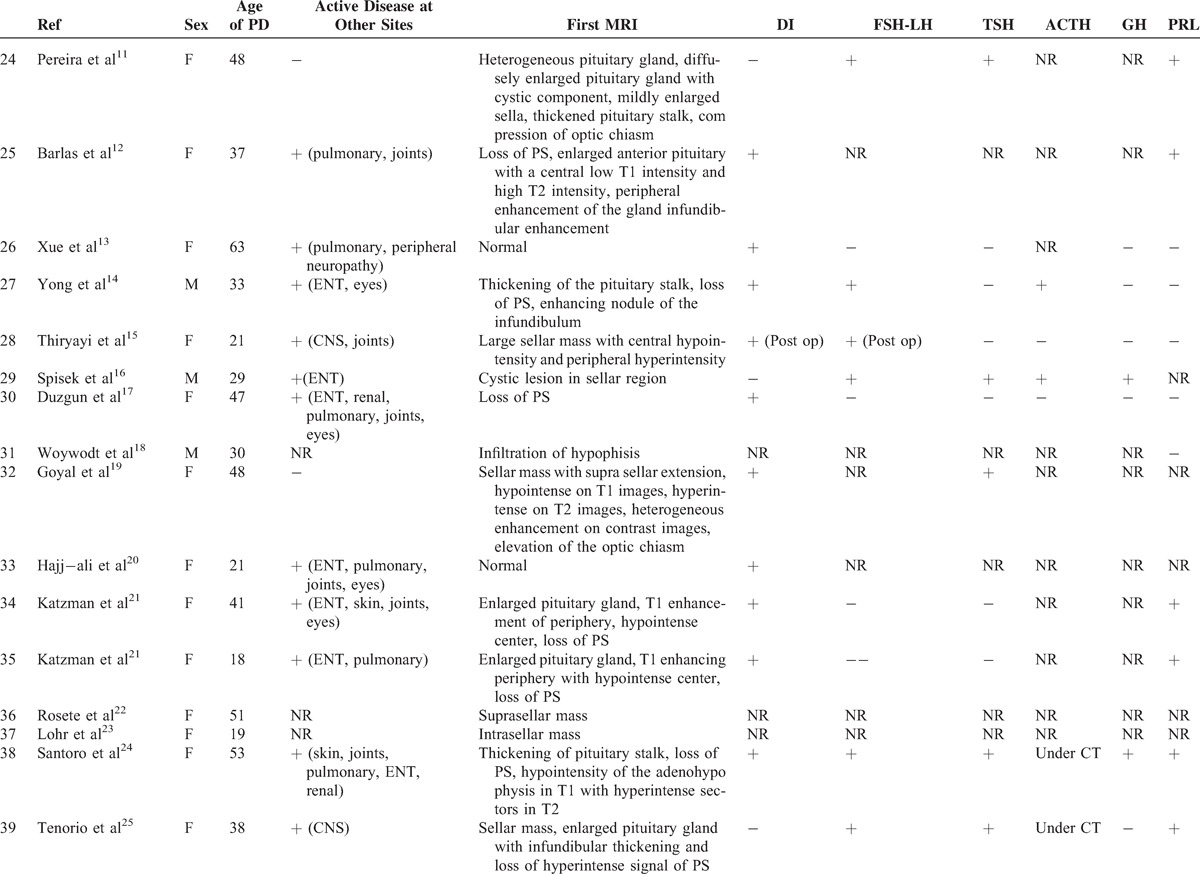
Clinical, Hormonal, Radiologic Features in patients With HP Involvement in GPA (Literature Review)

**TABLE 3 (Continued):**
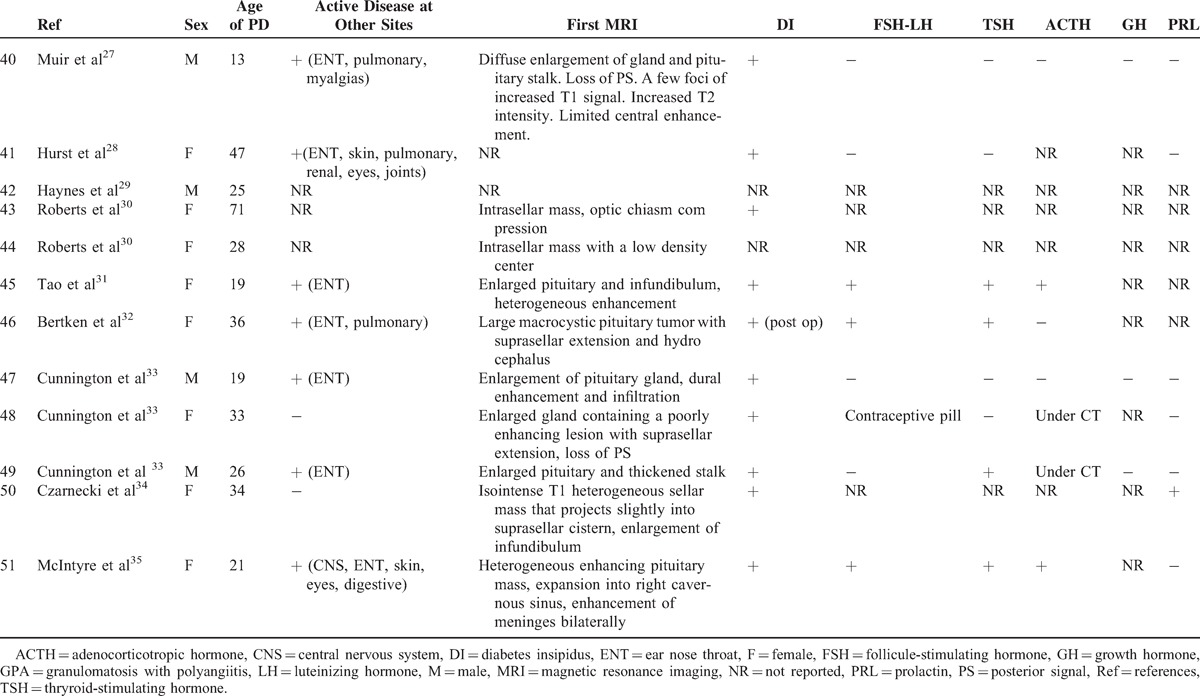
Clinical, Hormonal, Radiologic Features in patients With HP Involvement in GPA (Literature Review)

**TABLE 4 (Continued):**
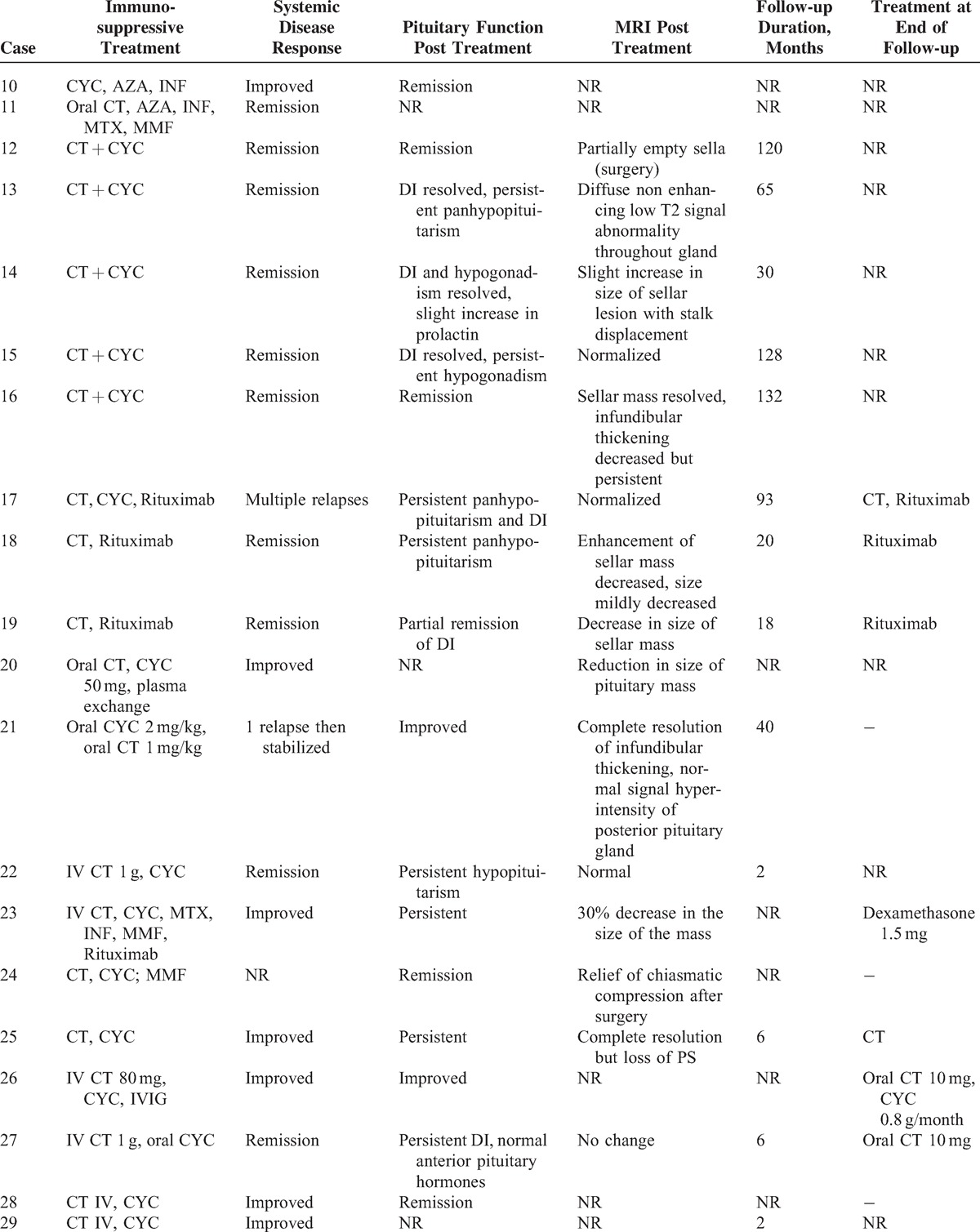
Treatment and Outcome of HP Involvement in Patients With GPA (Literature Review)

**TABLE 4 (Continued):**
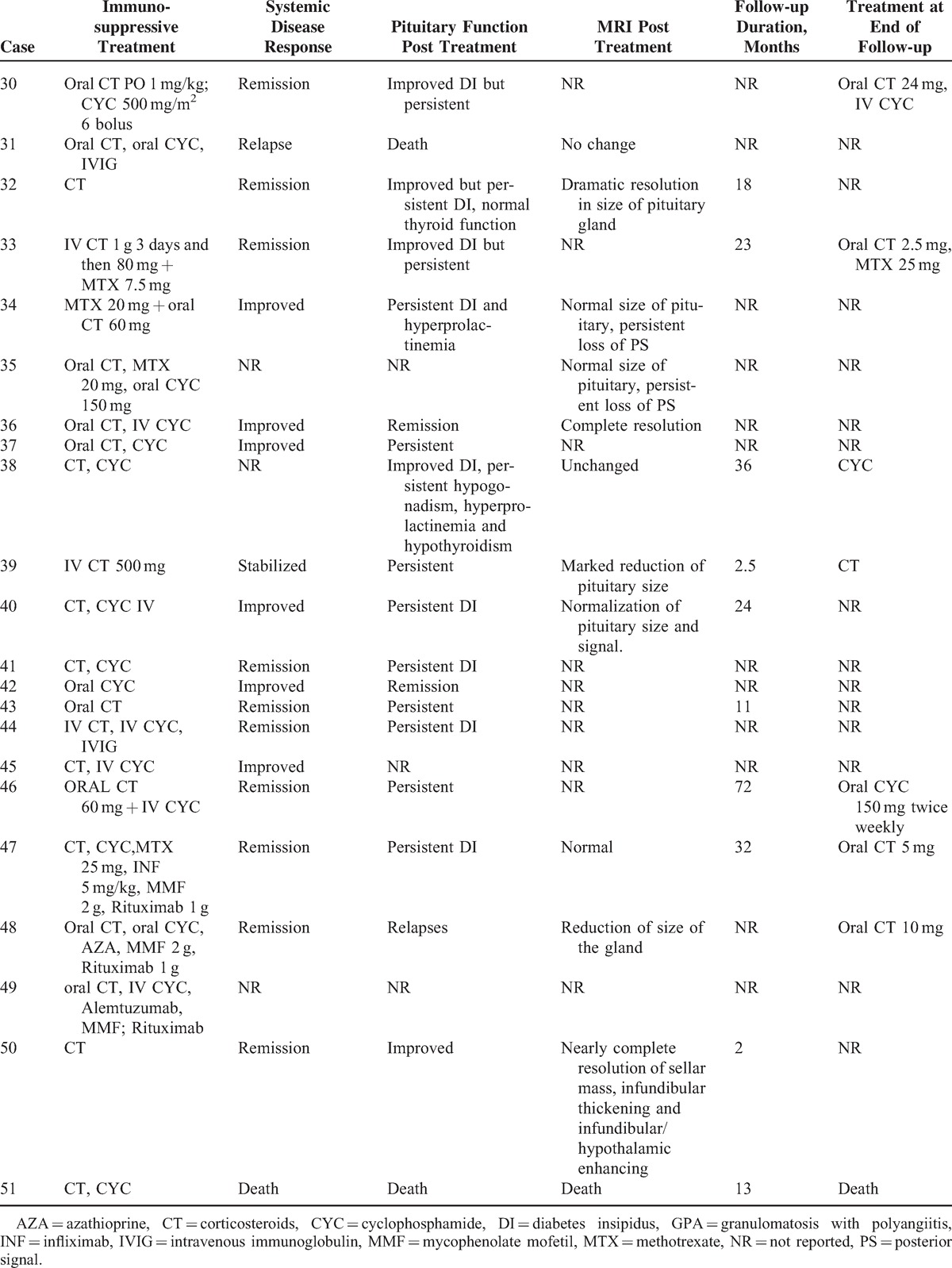
Treatment and Outcome of HP Involvement in Patients With GPA (Literature Review)

An anterior PD alone was reported in 6 patients and a posterior PD alone in 9 patients. Nineteen patients had both anterior and posterior PD and 1 patient had panhypopituitarism. The most common endocrine abnormalities were DI (n = 29) and hypogonadism (n = 19) followed by hypothyroidism (n = 15), hyperprolactinemia (n = 9), ACTH deficiency (n = 6),, and GH deficiency (n = 3). Among the 9 patients with hyperprolactinemia, 7 had lesions of the stalk on MRI.

Pituitary MRIs were available for 40 patients. Abnormalities of the pituitary were present in 95% of cases. The most common lesions were: a sellar mass (n = 22), enlarged pituitary (n = 13), loss of posterior pituitary bright spot (n = 11), thickening of the stalk (n = 12), abnormal enhancement of the stalk (n = 3), and compression of the stalk (n = 2) or the optic chiasm with visual defects (n = 8).

All patients received corticosteroids (n = 40) and/or immunosuppressive agents (n = 38) for HP involvement including: cyclophosphamide (n = 33), rituximab (n = 7), methotrexate (n = 6), azathioprine (n = 3), mycophenolate mofetil (n = 6), infliximab (n = 4), IVIG (n = 3), plasma exchange (n = 1), and alemtuzumab (n = 1). After treatment, only 7 patients had normal pituitary function and 12 patients had improved pituitary function. Fifteen had persistent PD, 6 were nonreported, and 2 patients died. However, the systemic disease was in remission in 20 patients and improved in 13 patients. A subsequent MRI was available in 26 cases. Seven patients had complete resolution after medical treatment, 10 had partial resolution, 4 had no change, 4 underwent surgery, and 1 had increased lesions. Resolution of hormonal deficiencies and imaging findings did not always correlate. Thus, among the 15 patients who had persistent PD, 4/10 had a complete resolution of MRI abnormalities, 6/10 had a partial resolution, and 5 patients had no subsequent MRI. Among the 7 patients who had normal pituitary function, 1 had a complete resolution of MRI abnormalities, 1 had a partial resolution, 2 underwent surgery, and 3 had no subsequent MRI.

Time to diagnosis was available for 7 patients. The 2 patients who were diagnosed respectively 2 and 3 weeks after initial symptoms had an improved or a normal pituitary function after treatment. The others were diagnosed with a median of 2 years after initial symptoms (3 months–7 years). Among these patients, all but 1 had persistent PD. Therefore, shortening diagnostic delays and treating these patients early in the course of the disease may prevent irreversible damage.

## DISCUSSION

To our knowledge, the present study derived from a national database is the largest series of patients with PD in GPA. Our results confirm that hypogonadism and diabetes insipidus are the most frequently reported endocrine disorder and that MRI abnormalities disappear or improve under corticosteroid treatment, whereas most hormonal deficiencies are irreversible.

Although admittedly a rare localization of GPA, the actual frequency of PD in GPA is difficult to estimate because it can differ depending on whether pituitary involvement is defined according to symptoms, hormonal measurements, MRI, or histopathology findings. No study has routinely searched for this localization of GPA in a large group of patients. Kapoor et al^3^ has reported 8 well-documented PDs among 637 GPA patients (1.3%) recruited in a tertiary referral center between 1996 and 2011. This prevalence is similar to our frequency of 1.1 % in the French Vasculitis Study Group cohort.

Symptoms of PD may be nonspecific including asthenia, headaches, vomiting, or muscular atrophy, which can suggest corticosteroid side effects. Such a clinical presentation and the insufficient awareness of this rare localization could explain the delay in the diagnosis of pituitary involvement in GPA patients estimated at 10 months in our study. The mean age at the onset of PD tended to be higher in our work than in the literature review: 50.8 years versus 38.6 years. However, the median age of onset of GPA is usually higher than in this literature review (49 years).^36^ We observed a slight female predominance, whereas in the FVSG cohort, there was a male predominance (54%).

Three different pathogenic mechanisms have been suggested to explain pituitary involvement: vasculitis, granulomatous formation in the pituitary, and granulomatous extension from contiguous sites (ENT). Of the 8 patients who underwent a pituitary biopsy in the literature, 4 had granulomatous inflammation^3,7,11,15^ and the others had inflammatory infiltrates.^10,16,35^ In our study, pituitary lesions were more frequently associated with active involvement of CNS (33.3% vs 10%) and eyes (44.4% vs 28.5%) than in the most recent series of the literature,^2,37^ which suggest a contiguous extension of granuloma. Even though it is unusual, 2 cases have previously been reported in the literature with normal MRIs suggesting the role of pituitary vasculitis.^13,20^ Indeed in our study, 2 patients had normal MRIs, which was confirmed by a neuroradiologist.

Our study confirms that pituitary involvement can be present at the time of diagnosis, but the symptoms mostly occur during the course of previous GPA within a time interval from several months or years after diagnosis. In most cases, there are signs of active disease at other sites, but pituitary involvement can be isolated and occur despite good control of the disease in other organs (ie, patient 5). In our study, HP lesions were never isolated when present at onset of GPA. It may be difficult to diagnose pituitary GPA when lesions are initially isolated, which has been described in 3 patients (7.6%) reported in the literature.^7–9^

Hypogonadism and diabetes insipidus are the most frequent pituitary disorders reported in our study. These findings are in agreement with Kapoor et al's report.^3^ By combining our study and the literature review, the frequency of diabetes insipidus in pituitary involvement can be estimated at 71% (n = 36/51 cases). Gonadotropin deficiency affected 78% of our patients with pituitary dysfunction, which is much higher than the cumulative-frequency calculated from the literature review (n = 19/42, 45%). Hypothalamic or pituitary deficiency may occur in systemic diseases such as GPA. However, a decrease in GnRH secretion can be related to other mechanisms such as drugs including corticosteroids, acute illness, malnutrition, and hyperprolactinemia. Indeed, 3 of our patients with gonadotropin deficiency and 5 of the patients reported in the literature had hyperprolactinemia. Thus, the mechanisms of hypogonadism in GPA remain unknown and are probably multifactorial. The frequency of hypogonadism due to GPA alone in our study could therefore be overestimated. In the literature and in our study, the frequency of other deficiencies among patients with pituitary dysfunction could be estimated at 54% for the TSH deficiency (20/37), 37% for the hyperprolactinemia (13/35), 38.8 % for the ACTH deficiency (7/18), and 20% for the GH deficiency (5/25). The latter was probably underestimated due to the lack of dynamic tests and the poor sensitivity of IGF1. One case of panhypopituitarism was noted.

Most patients in our study and in the literature review had MRI abnormalities and the most frequent lesions were enlargement of the pituitary gland (28.5%) or pseudo adenoma (48.9%), loss of posterior hypersignal on T1-weighed images (30.6%), thickening or infiltrative lesion of the pituitary stalk (34.6%), and enhanced lesions by gadolinium of the pituitary gland (28.5%).

Concerning the PD GPA treatment, the available information is limited. In Kapoor et al's study,^3^ all the patients were treated for pituitary involvement, but only 2 out of 8 patients recovered completely from hormonal deficiencies. In our study, all patients received a corticosteroid therapy, and all but 1 received immunosuppressive drugs resulting in remission of the systemic disease in most cases. By combining our study and the literature review, 69% of the patients were treated with a cyclophosphamide-based regimen, with a relapse rate of the systemic disease of 11%, and a median follow-up of 58.8 months. Rituximab has been shown to be of benefit in severe GPA refractory to cyclophosphamide therapy, and it has recently been approved for the treatment of ANCA-associated vasculitides based on results from large randomized controlled trials.^38^ In our study and in the literature review, none of the 7 patients treated with rituximab recovered completely from PD. However, 24% of the patients treated with a cyclophosphamide-based regimen had a normal pituitary function (7/29). As previously discussed, PD in GPA appears to be associated with the granulomatous component of the disease. Rituximab appears to be less efficient in the granulomatous component of the disease (ie, pachymeningitis, orbit and tracheal involvement) than in the “vasculitis” component of the disease, which lead us to preferentially consider its use for refractory PD GPA.^39^

Despite a high rate of systemic disease remission on maintenance therapy (57.4%) or stabilization (31.9%), 86% of the patients were left with residual pituitary hormonal deficits. No correlation was found between hormonal, radiologic, and general outcome, as confirmed in 2 other studies.^3,14^ These data suggest that, as previously described in hypothalamo-pituitary sarcoidosis,^40^ the granulomatous infiltration may early induce direct and definitive damage of the pituitary cells.

There are several limitations to this study. First, this is a multicenter study and a retrospective analysis with possible biases including the estimated prevalence of pituitary involvement. As there was no systematic screening for pituitary dysfunction, its prevalence may have been underestimated. In addition, diagnosis and management were not standardized. Actually, 28.8% of the cases, by combining our study and the literature review, could not be evaluated for ACTH deficiency because of a corticosteroid treatment. However, most cases were referred to an endocrinology unit with a hormonal evaluation using the same base-line serum hormonal measurements.

In conclusion, HP involvement in GPA is rare. The most frequent symptoms (headaches and asthenia) besides DI are unspecific and therefore the diagnosis may be difficult. Therefore, the prevalence of the HP involvement is probably underestimated. There is no correlation between hormonal, radiologic, and systemic outcome. HP lesions are usually associated with active disease at other sites, especially ENT, eye, and CNS involvement. Although corticosteroid therapy and immunosuppressive drugs improve vasculitis activity, hormonal deficiencies persist most of the time as in sarcoidosis. An early diagnosis is essential as prompt initiation of definitive therapy could induce disease remission and recovery of pituitary dysfunction.
